# GPU implementation of photoacoustic short-lag spatial coherence imaging for improved image-guided interventions

**DOI:** 10.1117/1.JBO.25.7.077002

**Published:** 2020-07-25

**Authors:** Eduardo A. Gonzalez, Muyinatu A. Lediju Bell

**Affiliations:** a Johns Hopkins University, School of Medicine, Department of Biomedical Engineering, Baltimore, Maryland, United States; b Johns Hopkins University, Whiting School of Engineering, Department of Electrical and Computer Engineering, Baltimore, Maryland, United States; c Johns Hopkins University, Whiting School of Engineering, Department of Computer Science, Baltimore, Maryland, United States

**Keywords:** coherence-based beamforming, graphical processing unit, photoacoustic, real-time, visual servoing

## Abstract

**Significance:** Photoacoustic-based visual servoing is a promising technique for surgical tool tip tracking and automated visualization of photoacoustic targets during interventional procedures. However, one outstanding challenge has been the reliability of obtaining segmentations using low-energy light sources that operate within existing laser safety limits.

**Aim:** We developed the first known graphical processing unit (GPU)-based real-time implementation of short-lag spatial coherence (SLSC) beamforming for photoacoustic imaging and applied this real-time algorithm to improve signal segmentation during photoacoustic-based visual servoing with low-energy lasers.

**Approach:** A 1-mm-core-diameter optical fiber was inserted into *ex vivo* bovine tissue. Photoacoustic-based visual servoing was implemented as the fiber was manually displaced by a translation stage, which provided ground truth measurements of the fiber displacement. GPU-SLSC results were compared with a central processing unit (CPU)-SLSC approach and an amplitude-based delay-and-sum (DAS) beamforming approach. Performance was additionally evaluated with *in vivo* cardiac data.

**Results:** The GPU-SLSC implementation achieved frame rates up to 41.2 Hz, representing a factor of 348 speedup when compared with offline CPU-SLSC. In addition, GPU-SLSC successfully recovered low-energy signals (i.e., ≤268  μJ) with mean ± standard deviation of signal-to-noise ratios of 11.2±2.4 (compared with 3.5±0.8 with conventional DAS beamforming). When energies were lower than the safety limit for skin (i.e., 394.6  μJ for 900-nm wavelength laser light), the median and interquartile range (IQR) of visual servoing tracking errors obtained with GPU-SLSC were 0.64 and 0.52 mm, respectively (which were lower than the median and IQR obtained with DAS by 1.39 and 8.45 mm, respectively). GPU-SLSC additionally reduced the percentage of failed segmentations when applied to *in vivo* cardiac data.

**Conclusions:** Results are promising for the use of low-energy, miniaturized lasers to perform GPU-SLSC photoacoustic-based visual servoing in the operating room with laser pulse repetition frequencies as high as 41.2 Hz.

## 1 Introduction

Visual servoing^1^^–^^3^ is a promising approach for maintaining visualization of surgical tools during minimally invasive procedures and keeping track of the location of nearby anatomical targets within the body. This approach broadly refers to vision-based robot control, and the robot “vision” that we focus on in this paper is provided through photoacoustic images.^4^^,^^5^ Photoacoustic imaging is achieved by transmitting pulsed light to a structure of interest, which absorbs the light, undergoes thermal expansion, and generates an acoustic response that is received by a conventional ultrasound probe.^6^^–^^8^ This photoacoustic imaging technique was previously demonstrated for multiple applications that require surgery or interventions, such as visualization of brachytherapy seeds,^9^^,^^10^ intravascular imaging,^11^ cardiac catheter visualization,^5^ fetal surgeries,^12^ prostate surgeries,^13^ and endonasal surgeries.^14^^–^^16^ In these applications, structures of interest include blood vessels, nerves, drill tips, and catheter or needle tips.^5^^,^^17^^,^^18^ One or more optical fibers may be coupled to the tool, catheter, or needle tips to transmit the light pulses.^11^^,^^19^^–^^21^ Alternatively, a fiber or fiber bundle may be operated independently to provide photoacoustic-based anatomical guidance in the absence of surgical tools.^22^^–^^24^

**Fig. 1:**
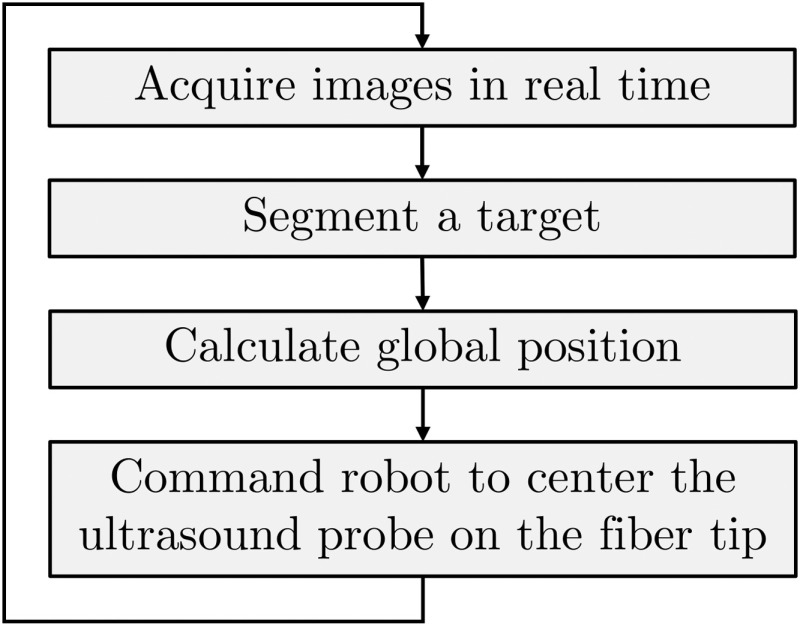
Overview of photoacoustic-based visual servoing.

With the rise of robotic surgery,^25^^–^^28^ we can reasonably envision photoacoustic system components that are robotically controlled to enable more successful surgeries and interventions.^29^ A summary of the procedures required to achieve photoacoustic-based visual servoing, in particular, is shown in Fig. 1, starting with a robot-held ultrasound probe that receives photoacoustic signals. Receive beamforming techniques are then applied to create a photoacoustic image, and an image segmentation algorithm locates features of interest within the image. Beamforming techniques are implemented rather than photoacoustic reconstruction techniques such as backprojection due to two considerations. First, beamforming is sufficient to accurately quantify the position and size of photoacoustic sources,^30^ which are two parameters of primary interest for photoacoustic-based visual servoing. Second, conventional linear or phased array ultrasound probes would be placed externally for the proposed visual servoing application, rather than the ring arrays (i.e., spherical or cylindrical detection surfaces) that are more favorable for backprojection algorithms and quantitative photoacoustic applications.^30^ After beamforming and image segmentation, the ultrasound probe motion is controlled by the robot to ensure that targets of interest remain at the center of the image.

Previous photoacoustic-based visual servoing studies have implemented conventional delay-and-sum (DAS) receive beamforming.^4^^,^^5^ However, DAS photoacoustic images contain poor signal-to-noise ratio (SNR) when using low laser energies, which compromises the performance of the segmentation step shown in Fig. 1. This limitation may be overcome by increasing the incident laser energy or otherwise enhancing the amplitude of photoacoustic signals. Although stable segmentation is often achieved with high laser energies, these energies tend to introduce side lobes and other artifacts. Therefore, alternative options for signal amplitude enhancement would be more suitable. For example, frame averaging enables the use of lower energies and simultaneously reduces incoherent noise and artifacts,^31^^,^^32^ but this option introduces motion artifacts,^33^^,^^34^ which negatively impact the overall accuracy of visual servoing.

Alternatives to DAS beamforming have demonstrated potential to overcome the limitations of poor target visibility without assistance from frame averaging. For example, minimum variance (MV) beamforming has been shown to suppress off-axis signals and improve spatial resolution by decreasing main lobe widths.^35^ However, MV beamforming is sensitive to sound speed changes and requires subarray averaging,^36^ more than one stage of MV calculations, or the combination of weighting factors.^35^^,^^37^ These additional steps increase the computational burden of this beamforming alternative. Similarly, a synthetic aperture focusing (SAF) approach is beneficial with regard to enhancing lateral resolution along the depth (or axial) dimension^38^^,^^39^ and reducing reverberation artifacts.^40^ However, these techniques require a combination of delay sequences for each pixel in the reconstructed image,^41^ which increases computational burden. Additional beamforming alternatives include coherence factor (CF) weighting^42^ or a combination of beamforming methods (e.g., DAS + CF,^43^ SAF + CF,^44^ and MV + CF^37^), and these options suffer from challenges similar to those stated above.

Short-lag spatial coherence (SLSC) beamforming^45^^–^^48^ is another option that has shown substantial promise in multiple interventional tasks.^9^^,^^15^^,^^16^^,^^29^^,^^49^ Therefore, SLSC is considered to be one of the more suitable beamforming options available to improve photoacoustic-based visual servoing. SLSC beamforming requires multiple normalized cross-correlations of delayed data to directly display measurements of aperture-domain spatial coherence rather than amplitude. Although SLSC beamforming is known to be insensitive to signal amplitude,^48^^,^^50^ the proposed application of visual servoing and surgical tool tracking does not require this sensitivity. The benefits of using SLSC beamforming for the proposed application are that it enhances the contrast of single-frame photoacoustic images (i.e., no frame averaging required)^15^^,^^46^^,^^51^ and triples effective penetration depths when compared with DAS beamforming.^9^^,^^46^ In addition, SLSC beamforming improves the quality of photoacoustic signals acquired with low laser energies,^52^ which is advantageous because the use of low laser energies can help to ensure laser safety. Miniaturized low-energy light delivery systems (such as pulsed laser diodes^52^^–^^54^ or light-emitting diodes^55^) are additionally beneficial for portability in the operating room and increasing frame rates when compared with Q-switched lasers. Therefore, we are interested in exploring capabilities and limitations of SLSC beamforming with regard to low-energy light sources.

Drawing on this history of promise and success, this paper extends our two previous conference papers,^56^^,^^57^ which describe elements of the first known real-time implementation of the SLSC beamformer for photoacoustic imaging, utilizing the graphical processing unit (GPU) of an FDA-approved joint clinical and research Alpinion E-CUBE 12R ultrasound system. The new contributions of this paper include a detailed assessment of the relationships among SLSC beamforming parameters, processing time, and image quality and reports of photoacoustic signal-to-noise ratios (SNRs) obtained with a range of laser energies, target depths, and SLSC beamforming parameters. This information is then used to evaluate the two essential visual servoing tasks of fiber tip tracking and probe centering and to compare these tasks with both the real-time SLSC beamformer and the DAS beamformer. Finally, our presented GPU-SLSC approach is evaluated with *in vivo* data.

This paper is organized as follows. Section 2 details the framework of our GPU-SLSC photoacoustic implementation and describes the methods used to assess performance. Section 3 demonstrates GPU-SLSC feasibility for real-time applications, as well as the improved SNR and increased tracking accuracy achieved with GPU-SLSC during visual servoing with low laser energies. Section 4 discusses these findings and their implications. Finally, Sec. 5 concludes the paper with a summary of the major technical contributions and achievements of this work.

## 2 Method

### 2.1 GPU Implementation of Photoacoustic SLSC Imaging

**Fig. 2:**
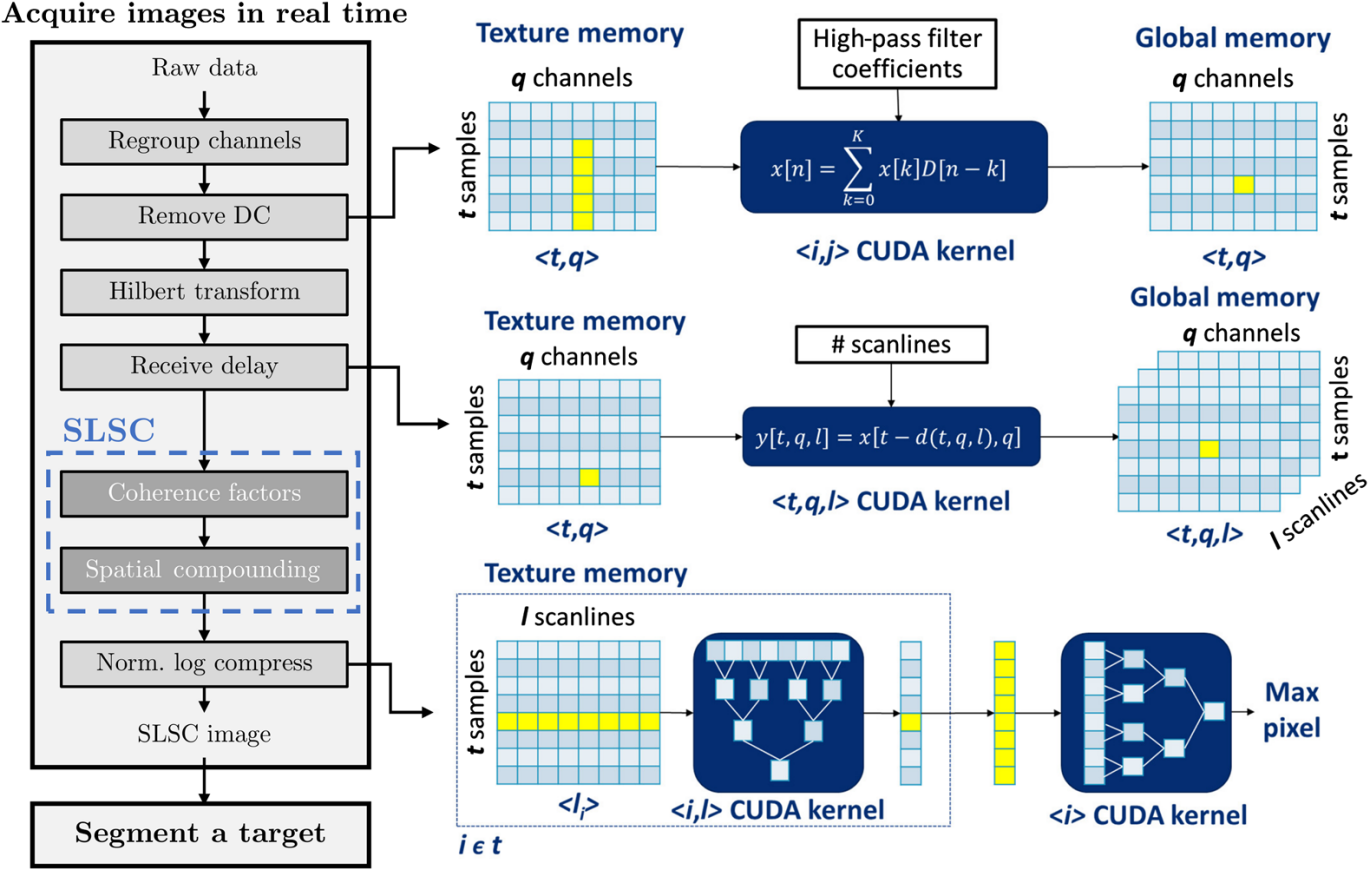
Workflow for acquiring a real-time photoacoustic SLSC image with the Alpinion ultrasound system. The diagrams on the right show graphical displays of GPU kernel distributions for the “Remove DC”, “Receive delay”, and “Norm. log compress” (normalize and log compress) steps of real-time photoacoustic SLSC imaging. The x, y, D, and d shown in the CUDA kernels denote the input memory, output memory, high-pass filter coefficients, and receive delays, respectively. Variables t, q, and l are indices for axial sample, channel, and scanline, respectively.

The steps to implement real-time SLSC imaging with photoacoustic data acquired with an Alpinion E-CUBE 12R system are shown in Fig. 2. First, raw channel data were acquired by the ultrasound system, which was triggered by a signal from the laser system. Depending on the ultrasound system memory allocation and the number of available channels, a regrouping process (i.e., “Regroup channels” in Fig. 2) was performed and transferred to the device texture memory as an $N_{i}\times N_{z}$ matrix, where $N_{i}$ is the number of elements and $N_{z}$ is the number of axial samples. The total number of acquisitions needed before processing the data is an integer computed as $N_{A}=N_{i}/N_{c}$, where $N_{c}$ is the number of channels.

The ordered raw data were stored in texture memory [i.e., read-only cached memory that optimizes physically adjacent two-dimensional (2-D) operations]. DC removal was then computed by applying one-dimensional convolutions of time-domain kernels, executed independently along the axial dimension. This operation is graphically displayed to the right of the “Remove DC” block in Fig. 2, showing operations at the compute unified device architecture (CUDA) kernel level when transitioning from texture memory to global memory (i.e., the general memory of the GPU device that lasts for the duration of the process). The Hilbert transform was then computed along the axial dimension using the fast Fourier transform (FFT) libraries embedded in CUDA (NVIDIA, Santa Clara, CA, USA). Here, FFT kernels ran independently across the element dimension $N_{i}$.

Next, synthetic receive aperture imaging was performed to generate a specific number of scanlines, $N_{x}$, determined by the user as an input parameter, obtaining an $N_{i}\times N_{x}\times N_{z}$ matrix. This operation is graphically displayed to the right of the “Receive delay” block in Fig. 2, showing operations at the CUDA kernel level when transitioning from texture memory to global memory. The computation of receive delays was performed in the device texture memory, optimized for 2-D linear interpolation, and kernels were distributed with a ratio of one thread per axial sample, executed independently across elements.

The SLSC computations were the same as those described for a comparative Verasonics ultrasound system implementation^58^ developed by Hyun et al.^59^ These processes are denoted as the dark gray boxes in Fig. 2. In contrast to the original SLSC implementation,^45^ the GPU approach computes a single ensemble correlation coefficient from an ensemble sum of coherence factors $C_{ij}$, $C_{ii}$, and $C_{jj}$ rather than an average over each coherence value from element pairs separated by a lag m, as described by the following equations:

$$C_{ij}(z,x,m)=\overset{N_{i}-m}{\underset{i=1}{\sum }}s_{i}(z,x)s_{i+m}{\left(z,x\right)}^{*},$$ (1)

$$C_{ii}(z,x,m)=\overset{N_{i}-m}{\underset{i=1}{\sum }}{\left|s_{i}(z,x)\right|}^{2},$$ (2)

$$C_{jj}(z,x,m)=\overset{N_{i}-m}{\underset{i=1}{\sum }}{\left|s_{i+m}(z,x)\right|}^{2},$$ (3)

where $s_{i}(z,x)$ is a complex signal at element i, scanline x, and axial sample z, and * denotes the complex conjugate. The coherence factors ($C_{ij}$, $C_{ii}$, and $C_{jj}$) were stored in the device global memory and then compounded across an axial kernel size, k, and a cumulative lag, M, which is defined as the cumulative sum up to the first M lags, as follows:

SLSC(z,x)=∑m=1M∑z^∈kCij(z^,x,m)∑z^∈kCii(z^,x,m)∑z^∈kCjj(z^,x,m). (4)

Finally, the negative SLSC values were set to zero,^60^ and the SLSC image was then normalized and log-compressed. The maximum term for normalization was computed using logarithmic reduction strategies.^61^ A graphical representation of the logarithmic reduction is shown to the right of the “Norm. log compress” block in Fig. 2 for two consecutive CUDA kernels. The first CUDA kernel computed the maximum value across the lateral dimension given a specific depth. This computation was performed by calculating a vector of maximum values from a layer of element pairs. The vector of maximum values was then distributed to a smaller layer of element pairs until a single maximum remains. The maximum value across the lateral dimension was stored in an axial vector, where a second CUDA kernel calculated the maximum value of the image with the same steps as the first CUDA kernel.

The entire GPU-SLSC photoacoustic implementation shown in Fig. 2 was executed on a GeForce GTX 1080 GPU (NVIDIA Corporation, Santa Clara, CA, USA), with 8 GB of Video Random Access Memory and a core clock speed of 1733 MHz. This GPU was installed on the Alpinion E-CUBE 12R ultrasound research system.

### 2.2 Processing Time Assessments

Processing times of the GPU-SLSC photoacoustic implementation were assessed as functions of beamforming parameters, M and k, and as a function of the overall image depth, d. M was varied from 5 to 35 in increments of 5, k was evaluated as 3, 11, 19, and 31 axial samples, and d was evaluated as 5- and 15-cm axial depths. An axial depth of 15 cm was evaluated as a worst-case scenario in which memory transfer between the central processing unit (CPU) and GPU would limit real-time imaging capabilities.

To provide computation time measurements that are not limited by the laser pulse repetition frequency (PRF) (i.e., 10 Hz), the external trigger from the laser (needed for synchronization of the laser and ultrasound systems to perform photoacoustic imaging) was disabled. Although no synchronization between the laser system and ultrasound system results in meaningless photoacoustic data, this absence of synchronization does not affect algorithm processing times nor the speed of the GPU-SLSC algorithm. In the absence of wait times for synchronization, each acquisition and beamforming process was performed immediately after the previous frame was displayed on the ultrasound software of the Alpinion E-CUBE 12R. The inverse of the frame rate displayed with the laser trigger disabled was reported as the processing time estimate of the real-time GPU-SLSC algorithm. Robustness in the estimation of computation times was achieved by averaging 10 readings of the frame rate obtained over a time span of 10 s.

In addition to measuring overall processing times, the processing time for each stage of the flow diagram shown in Fig. 2 was measured for the GPU and CPU versions of SLSC beamforming (with the selected optimal values of M, k, and d defined in more detail in Sec. 2.3). CPU-SLSC computations were conducted in a MATLAB environment using the host CPU of the Alpinion E-CUBE 12R system, which is an Intel Xeon E5-1620 with a 3.5-GHz clock speed and 32-GB RAM. The processing times from 10 CPU-SLSC executions were averaged to achieve robust estimates.

### 2.3 Image Quality Assessments

**Fig. 3:**
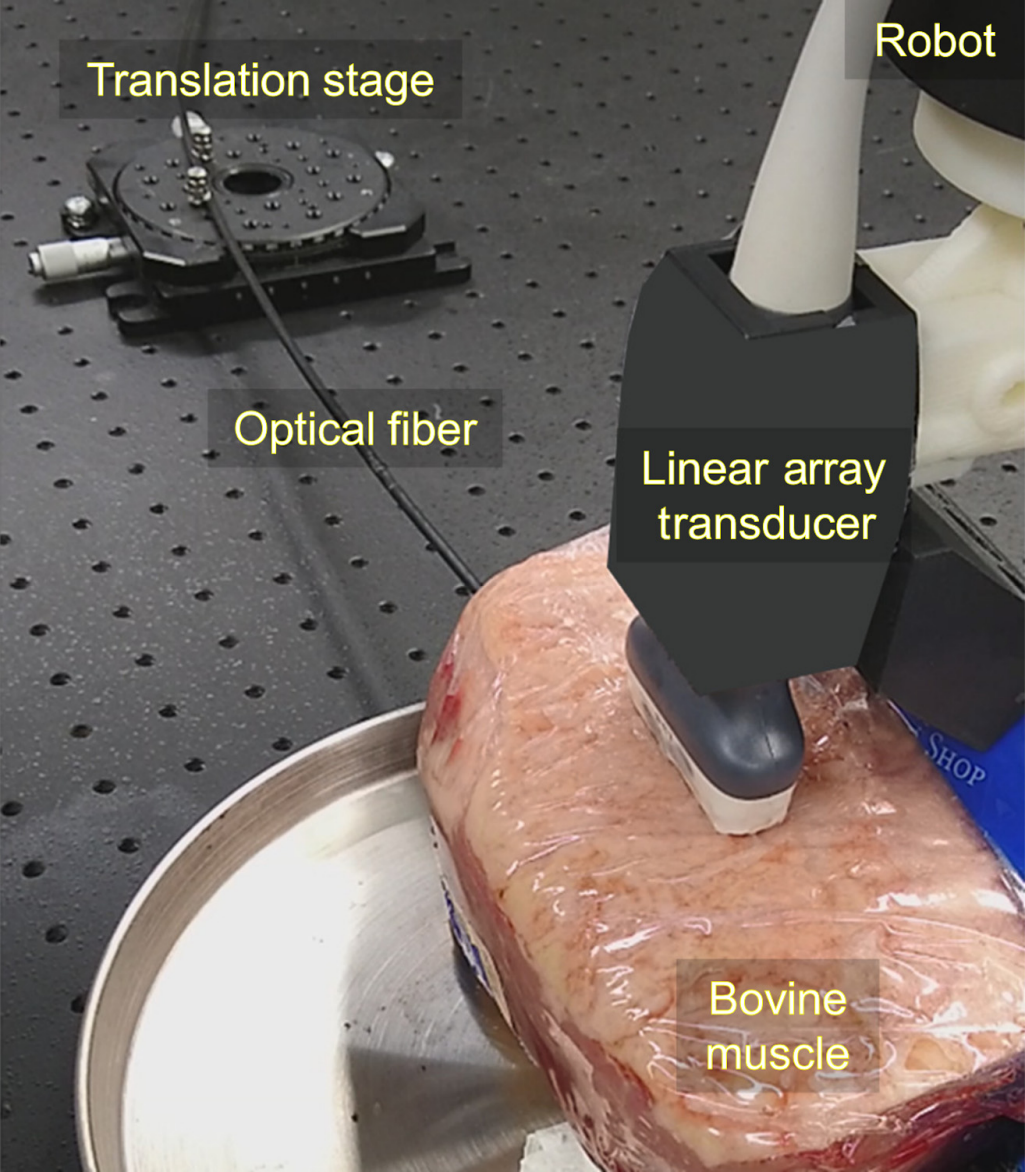
Photoacoustic acquisition setup. An optical fiber was attached to the translation stage and inserted into *ex vivo* bovine tissue. As the optical fiber was translated, an ultrasound probe connected to a robot arm performed visual servoing.

The experimental setup to assess image quality consisted of photoacoustic signals originating from an optical fiber tip inserted in *ex vivo* bovine muscle. The optical fiber was used to transmit 900-nm wavelength laser light from a Phocus Mobile laser (Opotek Inc., Carlsbad, CA, USA) with an energy of 726  μJ at the fiber tip. Photoacoustic signals were received by an L3-8 linear array ultrasound probe that was attached to a Sawyer robot arm (Rethink Robotics, Boston, MA), as shown in Fig. 3. To incorporate the effects of acoustic scattering and the expected depth-dependent image degradation, the optical fiber tip was located at depths of s=4  cm and s=7  cm. Considering that the primary source of photoacoustic signals is expected to originate from the tip of the fiber in interventional applications,^5^ we did not attach the fiber to any surgical tools in this study.

The overall image depth, d, was adjusted based on the target depth (i.e., d=5  cm when s=4  cm, and d=10  cm when s=7  cm). M and k were additionally varied for each target depth using the same ranges and increment sizes described in Sec. 2.2 (i.e., M was varied from 5 to 35 in increments of 5 and k was evaluated as 3, 11, 19, and 31 axial samples). The selection of optimal M and k values for these experiments was obtained by implementing three optimization criteria: (1) maximizing the differentiation between photoacoustic signals and background noise, (2) minimizing side lobes, and (3) minimizing temporal resolution (i.e., processing times).

The generalized contrast-to-noise ratio (gCNR)^62^^–^^64^ was used to assess the likelihood of discrimination between regions of interest (ROIs) of beamformed photoacoustic data and after normalization but before the log compression stage (i.e., the first optimization criterion for parameter selection).

$$\mathrm{gCNR}=1-\overset{1}{\underset{x=0}{\sum }}\underset{x}{\min}\{p_{i}(x),p_{o}(x)\},$$ (5)

where $p_{i}$ and $p_{o}$ are the probability density functions of signal amplitudes within ROIs inside and outside the target, respectively. The probability density functions were calculated from histograms computed with 256 bins. The inside ROI was a 3  mm×3  mm rectangle centered on the target center, which was defined as the brightest pixel within the photoacoustic image. The outside ROI was the same size and shifted 5 mm to the right of the lateral center of the target.

The lateral width of the photoacoustic target, $r_{\Delta }$, was assessed to quantify the extent and minimize the presence of side lobes (i.e., the second optimization criterion for parameter selection). This assessment was obtained by measuring the full width at half maximum (FWHM) of line plots passing through the center of the photoacoustic target, defined as

$$r_{\Delta }=\mathrm{FWHM}.$$ (6)

To determine the minimum possible energy limits for SLSC and DAS beamforming, the same experimental setup shown in Fig. 3 and described above was implemented with a shallower target depth of s=2.5  cm. Although the real-time parameters were optimized for deeper depths, it is reasonable to assume that similar or better image quality will be achieved at shallower depths. Given the 1-mm-core-diameter optical fiber geometry and the current standards for skin,^65^ the maximum permissible exposure (MPE) was $50\;\mathrm{mJ}/{\mathrm{cm}}^{2}$. This MPE translates to a maximum energy safety limit of 394.6  μJ. The laser energy was varied relative to this MPE (i.e., laser energies of 118, 184, 268, 364, 463, 570, and 645  μJ) for multiple photoacoustic image acquisitions.

The resulting SNR for each laser energy was evaluated with real-time SLSC and offline DAS beamforming as follows:

$$\mathrm{SNR}=\frac{\mu_{i}}{\sigma_{o}},$$ (7)

where $\mu_{i}$ is the mean value within an ROI of beamformed photoacoustic data inside the target (after normalization but before the log compression stage) and $\sigma_{o}$ is the standard deviation within an ROI of beamformed photoacoustic data outside the target (after normalization but before the log compression stage). The ROI for the signal was manually defined as a rectangle of approximately 2.5  mm×2.5  mm, centered on the target. Five independent background ROIs of the same size were placed 10 to 15 mm to the left of the lateral center of the target to obtain 5 SNR measurements that were used to report the mean ± standard deviation of SNR measurements. For each laser energy, SNR differences between DAS and either CPU-SLSC or GPU-SLSC, as well as SNR differences between CPU-SLSC and GPU-SLSC, were each evaluated using a repeated-measure analysis of variance to determine statistical significance (p<0.05).

### 2.4 Application to Visual Servoing

The visual servoing process (outlined in Fig. 1 and detailed as the velocity-based visual servoing procedure reported in our previous publication^5^) initiated with the acquisition of a real-time photoacoustic image that was then sent to a postprocessing algorithm for target detection. Fast computation and transferring of the photoacoustic image is a critical component of the visual servoing algorithm to avoid bottlenecks and to enable smooth ultrasound probe motions. Morphological operations such as dilation and erosion were then performed on the beamformed photoacoustic image to detect a single connected component and calculate its centroid. The lateral position of the centroid $\vec{p}$ was then saved and compared with the lateral center line of the image ${\vec{p}}_{0}$. The lateral difference was similarly computed ($\Delta \vec{p}=\vec{p}-{\vec{p}}_{0}$). Finally, the ultrasound probe was positioned with the goal of minimizing $\Delta \vec{p}$, effectively centering the ultrasound probe on the fiber tip.

Two visual servoing experiments were conducted in the *ex vivo* bovine muscle to assess the performance of visual servoing with real-time SLSC (i.e., GPU-SLSC) and real-time DAS beamforming. The first visual servoing experiment consisted of a probe centering test.^4^ During the initialization of this experiment, the probe was placed on the surface of the bovine tissue, and the length of the optical fiber was aligned with the imaging plane (i.e., $\Delta \vec{p}=0$). Then, the tip of the optical fiber was laterally displaced 6 mm from the center of the image. Visual servoing was deployed with the goal of ensuring that the final position of the lateral center of the ultrasound probe coincided with the segmented location of the fiber tip.

The second visual servoing experiment was performed after the ultrasound probe was centered. This experiment tested the ability of the visual servoing system to follow the fiber tip over a total distance of 10 mm, using a translation stage to achieve fiber advancement in tissue and to obtain ground truth displacement measurements. We refer to this second experiment as the fiber tracking experiment. The two visual servoing experiments (i.e., probe centering and fiber tracking) were performed with laser energies of 169, 248, and 322  μJ, which were lower than the maximum energy required to achieve laser safety with our system configuration (i.e., 394.6  μJ at the fiber tip, as described in Sec. 2.3). The statistical significance of performance differences between real-time SLSC and real-time DAS was evaluated with a Mann–Whitney U test.^66^

### 2.5 In Vivo Segmentation Assessment

Ideally, the experiments described in Sec. 2.4 would be repeated in an *in vivo* setting. However, the use of low laser energies was difficult to detect with DAS beamforming and repeated fiber tracking, and probe centering experiments were anticipated to unnecessarily extend the duration of an *in vivo* study, potentially causing unnecessary animal discomfort.

Therefore, we implemented an alternative plan. The segmentation performance with DAS, CPU-SLSC, and GPU-SLSC imaging was tested with *in vivo* data obtained from a previously completed experiment consisting of an optical fiber inserted in a porcine heart, as described in more detail in our previous publication.^5^ To summarize the data acquisition procedure, the optical fiber was first inserted into a cardiac catheter, and then the fiber–catheter pair was guided to the right atrium of the heart. The fiber emitted a laser wavelength of 750 nm with a pulse energy of 2.98 mJ. A total of 10 frames of resulting photoacoustic data were acquired with an Alpinion SP1-5 phased array ultrasound probe. This study was approved by the Johns Hopkins University Animal Care and Use Committee.

Considering the relatively high laser energy that was utilized in this previous experiment,^5^ Gaussian-distributed noise was added to the raw *in vivo* channel data as a surrogate for decreasing the laser energy. The resulting channel SNR of the raw *in vivo* data was evaluated as follows:

$${\mathrm{SNR}}_{c}=20\;\log_{10}(\frac{{\text{signal}}_{\mathrm{rms}}}{{\text{noise}}_{\mathrm{rms}}}),$$ (8)

where the rms refers to the root-mean-square of either the signal or the noise. The signal was defined as the entire channel data recording, and the noise was defined as the Gaussian random matrix added to the channel data. The channel data with added noise were then beamformed using DAS, CPU-SLSC, and GPU-SLSC, and photoacoustic signals were segmented using the algorithm described in our previous publication.^5^ The locations of the segmented signals were compared with ground truth segmentations derived from corresponding *in vivo* channel data with no noise added. A failed segmentation was defined as either no segmentation result or no overlap of the segmented signal with the ground truth segmentation.

## 3 Results

### 3.1 Selection of Beamforming Parameters

**Fig. 4:**
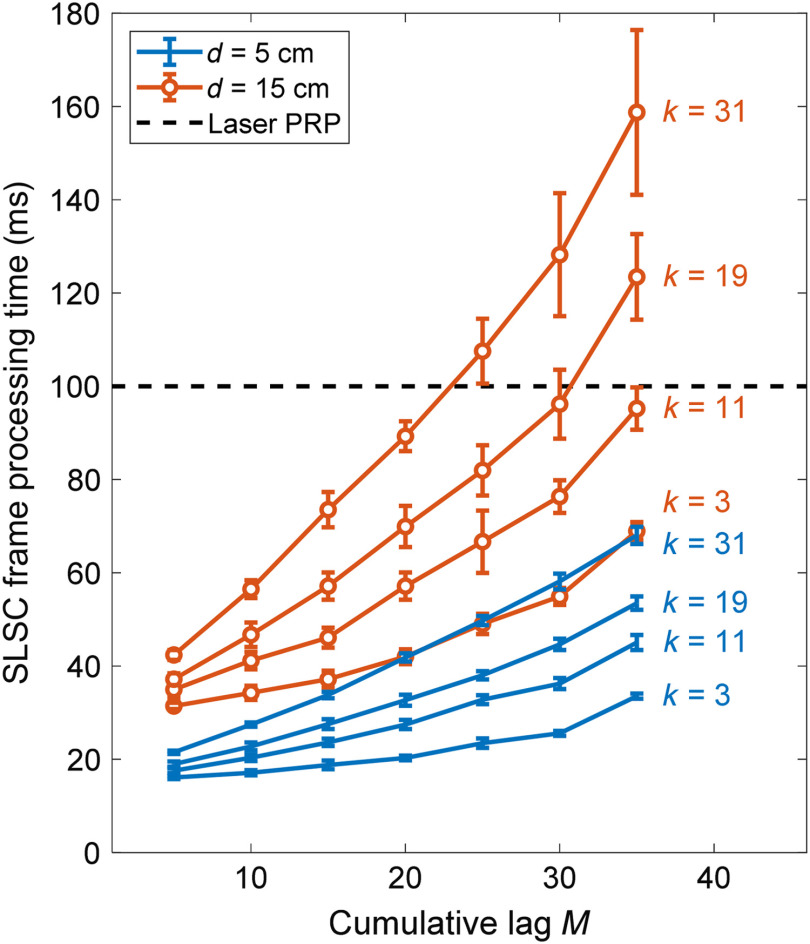
GPU-SLSC processing times for a single image frame, acquired with d=5  cm and d=15  cm imaging depths while varying the cumulative lag M and axial kernel size k.

Figure 4 shows GPU-SLSC processing time results for several pairs of M and k at two imaging depths. These processing times are limited by the maximum pulse repetition period (PRP) of any laser, which equals 100 ms for our laser, which has a 10-Hz PRF. Ideally, the processing times would be maintained below this limit (indicated by the dashed line) to avoid bottlenecks due to signal processing.

At 5-cm image depth, GPU-SLSC imaging reconstructs frames below this limit. At 15-cm image depth (which represents a worst-case scenario for internal light delivery with external ultrasound probe placement^9^^,^^15^^,^^46^^,^^49^^,^^67^), the majority of possible M and k parameters also fell below this limit. Specifically, pairs of [M>20, k≥31] and [M>25, k≥19] resulted in processing times above the laser PRP indicated by the dashed line. In addition, the observed increase in standard deviation as M increased is proportional to the increased number of operation loops associated with the CUDA kernels.

**Fig. 5:**
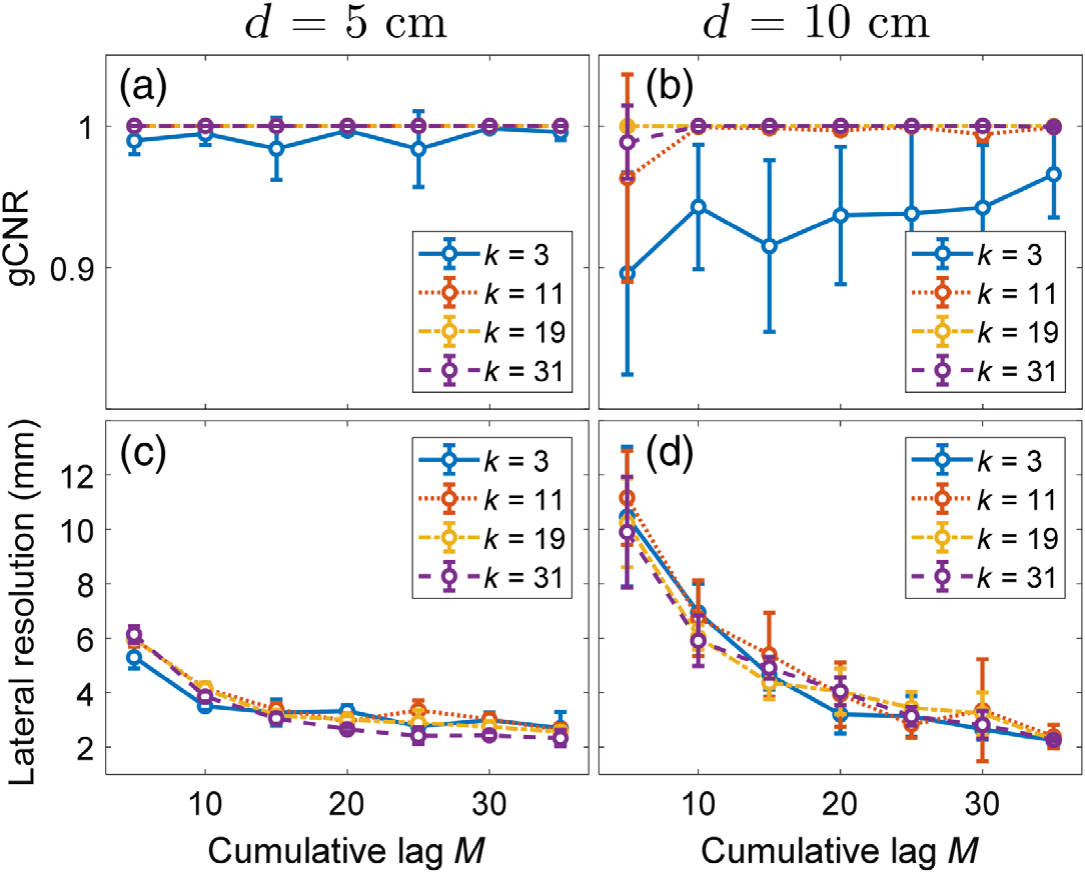
Image quality comparisons of (a, b) gCNR and (c, d) lateral resolution as functions of cumulative lag and axial kernel size for imaging depths of (a, c) d=5  cm and (b, d) d=10  cm.

Figure 5 shows image quality metrics as functions of M, k, and d. The discrimination between the source and the background provided by the gCNR metric [Figs. 5(a) and 5(b)] is the worst for both depth values when k=3, although the gCNR is generally good in most of these cases. The mean ± one standard deviation of gCNR values shown in Fig. 5 is 0.97±0.02 and 0.89±0.70 when d=5  cm and d=10  cm, respectively. When d=10  cm, the lowest M and k values in Fig. 5(b) show decreased gCNR, and gCNR is otherwise constant as M increases. Figures 5(c) and 5(d) show that lateral resolution improves as M increases, which is expected.^9^^,^^45^^,^^48^

The optimal M and k values were selected based on our observations of Figs. 4 and 5. Specifically, lateral resolution improvement was minimal when M>25 (Fig. 5) and temporal resolution generally remained below the 100-ms PRP limit at M=25 (Fig. 4). Therefore, M=25 was selected as optimal. The optimal k value was selected by maximizing gCNR, considering that successful discrimination between target and background is critical for the visual servoing segmentation algorithm. A value of k=11 was chosen because this value results in a gCNR of approximately 1 and increasing k beyond this value is expected to decrease temporal resolution and axial resolution, as previously reported for ultrasound SLSC implementations.^59^^,^^68^

### 3.2 Speedup of GPU-SLSC Compared with CPU-SLSC

**Fig. 6:**
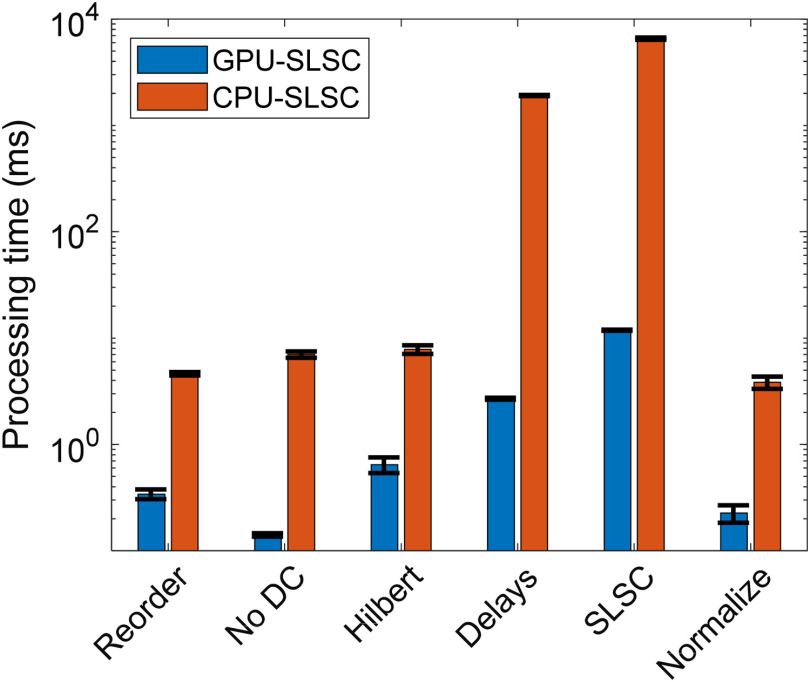
Processing times for each stage of SLSC beamforming using GPU and CPU implementations.

A depth of d=5  cm and the optimal values determined in Sec. 3.1 (i.e., M=25 and k=11) were implemented to compare computation times. The size of the raw data was a 3328×128 matrix of 16-bit resolution for this evaluation. Figure 6 shows the average processing times for each stage of the SLSC beamforming flow diagram with GPU-SLSC and CPU-SLSC implementations. GPU-SLSC imaging reduced computation times for each of the processing stages in comparison to CPU-SLSC imaging, with speedups of 13.5×, 49.7×, 12.1×, 711.2×, 550.9×, and 16.9× for the “Reorder,” “No DC,” “Hilbert,” “Delays,” “SLSC,” and “Normalize” stages, respectively.

Comparing the sum of the processing times (i.e., 15.91 ms) with the computation time between frames (i.e., 24.3 ms) resulted in a measured overhead of 8.39 ms. This overhead, likely due to memory transfer and intrinsic subroutines of the ultrasound software of the Alpinion E-CUBE 12R, was included when assessing the overall performance of our real-time implementation. With this inclusion, GPU-SLSC provided an overall speedup of 348.7× when compared with CPU-SLSC. Translating the computation time to real-time imaging scenarios, GPU-SLSC enabled a frame rate up to 41.2 Hz.

### 3.3 Performance in Ex Vivo Tissue

**Fig. 7:**
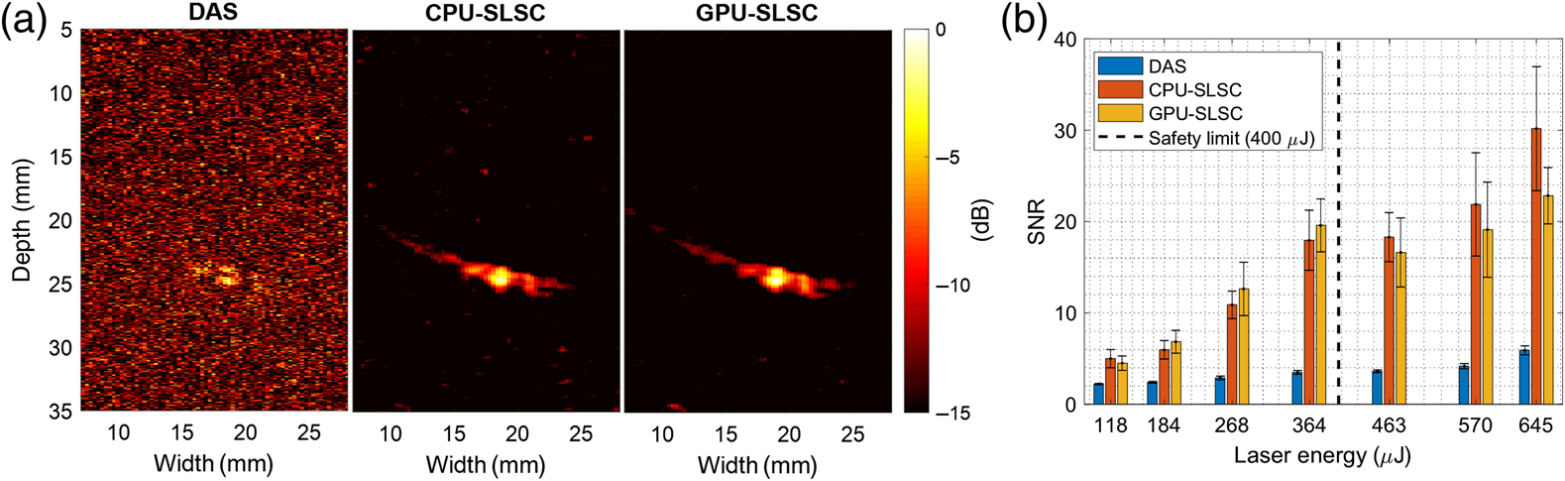
(a) Photoacoustic images of the optical fiber inserted into *ex vivo* bovine muscle at 25 mm axial depth, operating at 268 μJ laser energy. Images were reconstructed with DAS, CPU-SLSC, and GPU-SLSC beamformers. (b) SNR results from the optical fiber inserted into *ex vivo* bovine muscle as a function of the laser energy.

Figure 7 shows examples of beamformed photoacoustic images of the fiber tip acquired with a laser energy of 268  μJ [Fig. 7(a)] along with SNR measurements as a function of laser energy [Fig. 7(b)]. The SLSC and DAS images in Fig. 7(a) were normalized, log-compressed and displayed with a dynamic range of 15 dB. The mean ± one standard deviation of the SNR measured in the DAS, CPU-SLSC, and GPU-SLSC images of Fig. 7(a) were 3.5±0.9  dB, 11.4±2.9  dB, and 12.1±4.2  dB, respectively. As illustrated in Fig. 7(a), SLSC imaging consistently outperformed DAS imaging and visualized low-energy signals (≤268  μJ) with a mean SNR of 11.2±2.4 (p<0.05) The corresponding DAS SNR was 3.5±0.8. The mean SNR difference between the CPU-based and GPU-based SLSC implementations was 1.14. This difference was not statistically significant (p>0.05).

**Fig. 8:**
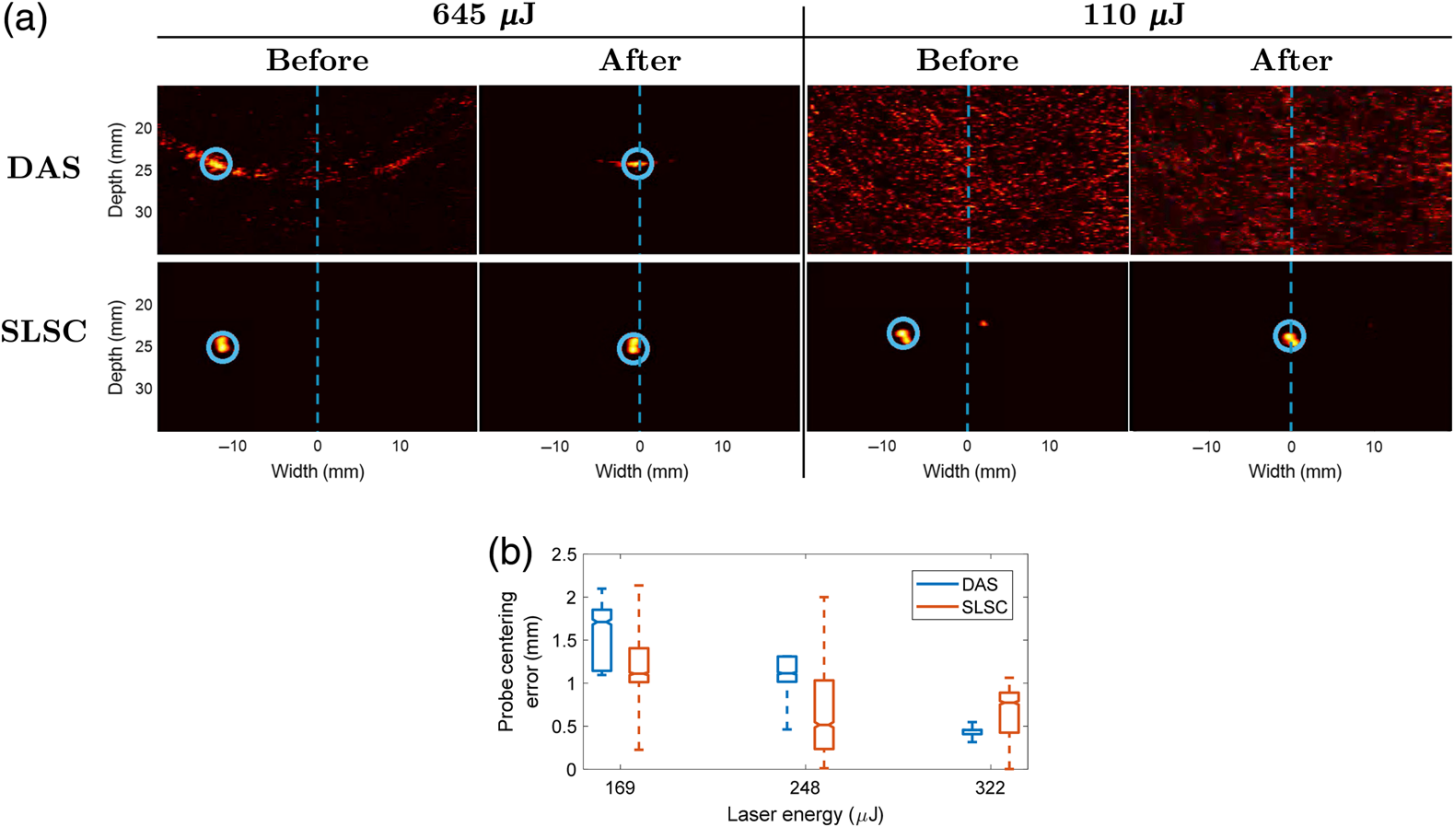
(a) Example of probe centering results for high and low laser energies with DAS and GPU-SLSC photoacoustic images. The first and second columns for each energy show the initial and final position of the fiber before and after probe centering, respectively. The dashed blue line represents the center of the image. The blue circle denotes the target detected by the segmentation algorithm. Video 1 contains a real-time display of these results, including additional photoacoustic images acquired between the before and after still frames (Video 1, MP4, 8.34 MB [URL: https://doi.org/10.1117/1.JBO.25.7.077002.1]). (b) Probe centering experiment errors.

Figure 8 shows the results of the probe centering experiment. Photoacoustic images acquired with one low laser energy (i.e., 110  μJ) and one higher laser energy (i.e., 645  μJ) are shown in Fig. 8(a). For the higher laser energy (which is higher than the safety limit of 394.6  μJ), visual servoing with either DAS or GPU-SLSC beamforming successfully accomplished the probe centering task. The left side of Fig. 8(a) shows the position of the fiber tip before and after the execution of visual servoing. The segmented target (denoted by the blue circle) is present and constant in both DAS and SLSC images at 645  μJ. The right side of Fig. 8(a) shows a corresponding result for the lower laser energy. While visual servoing with DAS generally failed to segment the target due to the low SNR, visual servoing with SLSC beamforming successfully performed the probe centering task.

Probe centering errors measured between desired centering locations and the actual robot positions are shown in Fig. 8(b) for three energies below the safety energy limit and within the range of energies shown in Fig. 8(a). These probe centering errors were obtained over a time period of 12 to 15 s after the first intersection of segmented target position with the center of the image. Five error measurements were computed for each energy. The horizontal line inside each box displays median error. The upper and lower edges of each box represent the first and third quartiles of the data set, respectively. The vertical lines connected to the boxes extend to the minimum and maximum values in each data set.

With 169-μJ laser energy, the median and interquartile range (IQR) of tracking errors were 1.71 and 0.71 mm, respectively, with DAS-based visual servoing and 1.11 mm and 0.39 mm, respectively, with SLSC-based visual servoing. Similarly, with 248-μJ laser energy, there was a higher median tracking error with DAS beamforming (i.e., 1.11 mm) than that obtained with SLSC beamforming (i.e., 0.52 mm). However, SLSC beamforming had a higher IQR of tracking errors (i.e., 0.79 mm) when compared with DAS beamforming (i.e., 0.29 mm) at the same laser energy. With 322-μJ laser energy, the median and IQR of tracking errors were 0.46 mm and 0.05 mm, respectively, with DAS-based visual servoing. These errors were lower than the median and IQR of tracking errors obtained with SLSC beamforming, which were 0.77 mm and 0.47 mm, respectively. Overall, for the three laser energies (i.e., 169, 248, and 322  μJ), the median and IQR of tracking errors were 1.10 mm and 0.85 mm, respectively, with DAS and 0.81 mm and 0.68 mm, respectively, with SLSC. For each laser energy in Fig. 8(b), the differences between the median probe centering error results with SLSC- and DAS-based visual servoing were statistically significant (p<0.01).

**Fig. 9:**
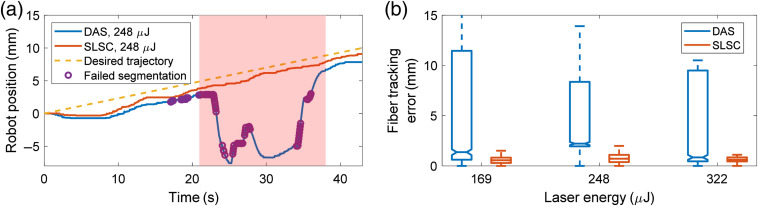
Fiber tracking results. (a) Example of robot positions with fiber tracking at 248  μJ. The circles represent time stamps when the visual servoing algorithm failed to segment the photoacoustic signal. (b) Fiber tracking errors at mid-range energies.

Figure 9 shows the results of the fiber tracking experiment. The trajectories of the robot-held ultrasound probe obtained during DAS- and SLSC-based visual servoing are compared with the desired trajectory in Fig. 9(a). Ideally, the trajectories generated with visual servoing would be closely related to the desired trajectory performed manually with the translation stage and indicated with the dashed line. Both DAS- and SLSC-based visual servoing followed the fiber displacement during the 0- to 18-s time interval. After 18 s, the noise present in the DAS image contributed to failed segmentations, resulting in a visual servoing failure, which is shown as the circles in Fig. 9(a).

When consecutive instances of failed segmentation were recorded over a 1-s time period, the robot performed a search around the current region in the lateral and elevation ultrasound probe directions. This searching algorithm was responsible for the increased deviation of the segmented target locations from desired locations with DAS-based visual servoing, as observed in the red shaded region of Fig. 9(a) (i.e., within the 21- to 38-s time interval). During this time interval, the median and IQR of tracking errors were 10.64 mm and 7.68 mm, respectively, with DAS-based visual servoing. When excluding this interval, the median and IQR of the difference between actual and desired trajectories were 2.02 mm and 0.41 mm, respectively, with DAS beamforming and 1.17 mm and 0.68 mm, respectively, with SLSC beamforming. Therefore, the real-time SLSC approach produced less deviations from the desired trajectory overall.

Tracking errors measured between the desired locations and the measured robot positions are summarized in Fig. 9(b). These errors were computed from visual servoing data obtained between two timestamps. The first timestamp was acquired when both the robot position and the desired location were initialized (i.e., $\overset{\to }{p}={\overset{\to }{p}}_{0}$, t=0). The second timestamp was acquired after the fiber was displaced by 10 mm with the translation stage. Generally, tracking errors were larger with DAS compared with SLSC beamforming for each laser energy shown in Fig. 9(b). Overall, for the three laser energies (i.e., 169, 248, and 322  μJ), the median and IQR of tracking errors were 2.01 mm and 8.97 mm, respectively, with DAS beamforming and 0.64 mm and 0.52 mm, respectively, with SLSC beamforming. For each laser energy, the differences between the median tracking error results with SLSC- and DAS-based visual servoing were statistically significant (p<0.01).

### 3.4 In Vivo Performance

**Fig. 10:**
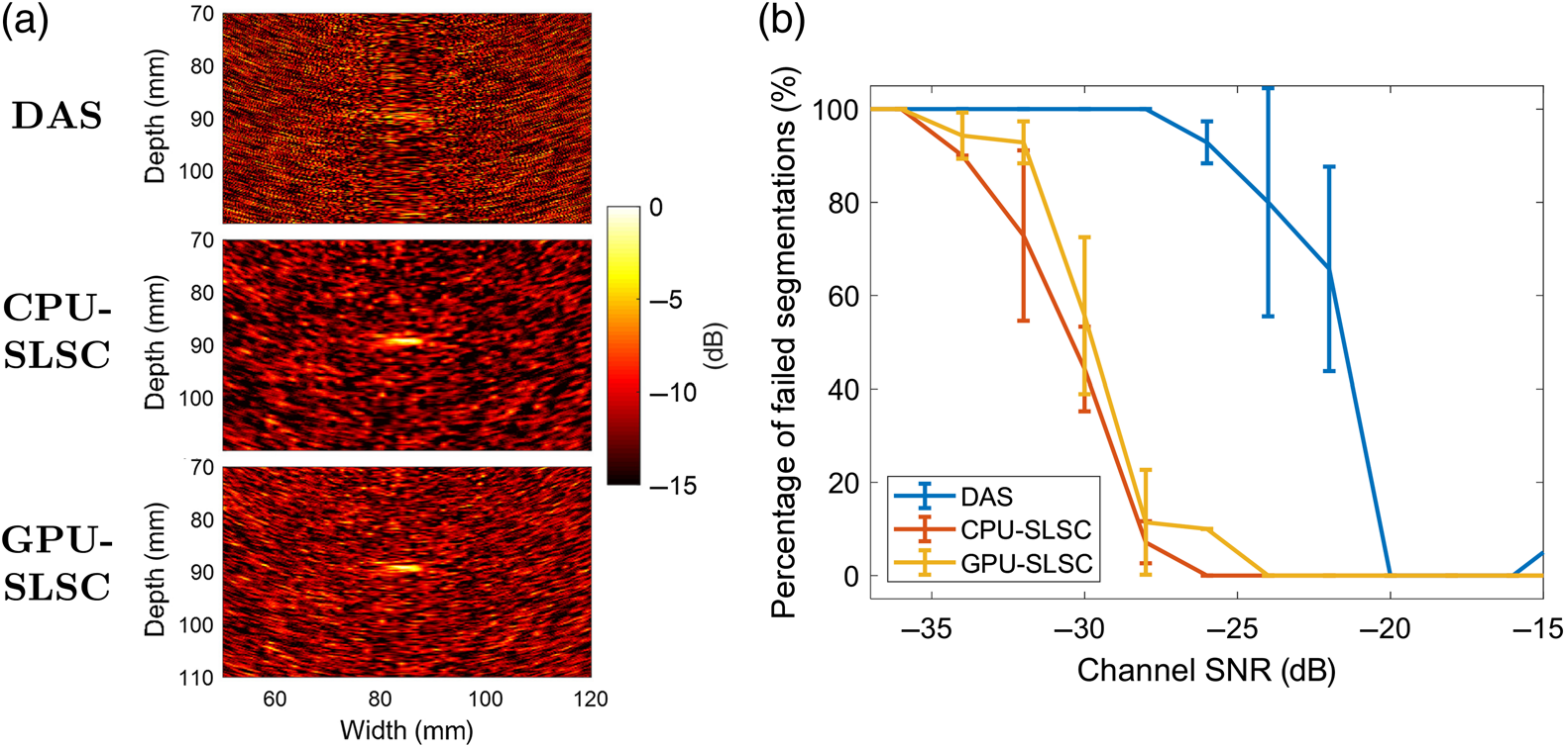
(a) Examples of *in vivo* DAS, CPU-SLSC, and GPU-SLSC photoacoustic images, created from the same raw data after adding Gaussian-distributed noise to achieve −30-dB channel SNR. The segmentation algorithm failed on the DAS image and succeeded with the CPU-SLSC and GPU-SLSC images. (b) For each channel SNR, the percentage of failed segmentations is represented as the mean ± one standard deviation of 10 measurements obtained after varying the amplitude threshold in the segmentation algorithm from 35% to 66% of the maximum amplitude within each photoacoustic image.

Figure 10(a) shows *in vivo* images created with DAS, CPU-SLSC, and GPU-SLSC beamformers after adding noise resulting in −30-dB channel SNR [i.e., ${\mathrm{SNR}}_{c}$ in Eq. (8)]. The percentage of failed segmentations measured from 10 frames of photoacoustic data is shown as a function of channel SNR in Fig. 10(b), represented as the mean ± one standard deviation of measurements obtained after varying the amplitude threshold in the segmentation algorithm from 35% to 66% of the maximum amplitude within each photoacoustic image. At −36-dB channel SNR, each beamforming method completely fails to segment the photoacoustic target (i.e., 100% failure). As channel SNR improved, the percentage of failed segmentations was reduced with CPU-SLSC and GPU-SLSC beamforming, measuring an average of 2.5% from −28- to −20-dB channel SNR. On the other hand, DAS beamforming resulted in a higher percentage of failed segmentations for the same range of channel SNRs. For channel SNRs>−20  dB, the noise levels were not sufficient to affect the segmentation performance of each beamformer, which is consistent with our observations at higher laser energies.

## 4 Discussion

Image-guided interventions often require visualization and tracking of important targets and structures. This requirement is well-suited to photoacoustic-based visual servoing of surgical tool tips. GPU-SLSC beamforming in particular provides three advantages to photoacoustic-based visual servoing in comparison with DAS and offline SLSC implementations. The first advantage is the reduction in computation times when compared with CPU-SLSC with minimal alterations to the precision of the coherence value estimations, as qualitatively observed in Fig. 7(a). The SNR difference between CPU-SLSC imaging and GPU-SLSC imaging is 1.14±3.99. Although minimal and not statistically significant, this difference is likely due to the single-precision libraries and linear interpolation in the GPU texture memory. Specifically, the GPU-SLSC algorithm utilizes CUDA embedded functions, such as the inverse square root, power, and cosine functions. These single-precision functions have unit in the last place errors of 9, 2, and 2, respectively,^69^ which decreases the precision of the coherence factors and the overall SLSC value. In addition, a GPU-SLSC imaging frame rate of 41.2 Hz, as described in Sec. 3.2, allows for visual servoing without overlapping subsequent acquisitions controlled by the laser trigger, which had a PRF of 10 Hz. After the SLSC image generation, a wait time of approximately 75  ms was required until the next trigger event, because raw data were acquired every 100 ms and the GPU-SLSC implementation with our 10 Hz laser generated a photoacoustic image every 24.3 ms.

The second advantage of GPU-SLSC beamforming is the improvement of photoacoustic signals at low energy levels, as shown in Fig. 7(b). We focus on low-energy lasers for two reasons. First, low laser energies enable miniaturization of the light delivery system. Second, low laser energies ensure minimal risk of damage to tissues for cases in which no safety limits currently exist (which is true for most tissues except for skin and eyes^65^). Although we can increase the laser energy, the presence of artifacts may be misinterpreted by the visual servoing algorithm if the energy is too high, and in other cases within the safety limit, a smaller increase in laser energy does not affect the GPU-SLSC visual servoing performance, as shown in Figs. 8(b) and 9(b). From this perspective, the focus of this work is targeted toward clinical applications that will require miniaturized systems with reduced risk of laser exposure for patients. With the experimental setup shown in Fig. 3, the laser safety limit for skin is 394.6  μJ, as determined by the diameter of the optical fiber and wavelength of excitation. An optical fiber with a smaller diameter would produce a lower safety limit at this same excitation wavelength (e.g., 142  μJ for a 0.6-mm-diameter optical fiber). Because GPU-SLSC can successfully recover signals obtained with energies as low as 118  μJ (particularly in cases in which DAS beamforming failed), our results indicate that GPU-SLSC will be beneficial in smaller and more portable light delivery systems,^52^ which is a necessary design requirement for effective visual servoing in the operating room.

The third advantage of GPU-SLSC beamforming is the robustness of SLSC-based visual servoing to segmenting and tracking signals at low (e.g., 110  μJ) and mid-range (e.g., 169 to 322  μJ) laser energies, when compared with photoacoustic-based visual servoing with DAS images. This robustness is particularly advantageous when considering that the visual servoing algorithm initiates a search process after a series of unsuccessful segmentation events are recorded.^5^ The probe centering and fiber tracking results [Figs. 8(a) and 9, respectively] indicate that the search process would be triggered more often with DAS than SLSC (see example provided in Video 1). This search motion would hinder effective tool tracking during surgery and add delays to the overall surgical or interventional procedure. GPU-SLSC beamforming has the potential to avoid these interruptions, which is additionally supported by the *in vivo* results shown in Fig. 10.

One detail that may be considered unsupportive of these three advantages of SLSC beamforming is the similar probe centering errors obtained at the mid-range energies shown in Fig. 8(b). However, DAS beamforming produced considerably higher fiber tracking errors than SLSC beamforming [i.e., the difference between the IQR of tracking errors shown in Fig. 9(b) was 8.45 mm]. Based on the combined outcome of these two experiments and the minimal overall percentage of failed segmentations with GPU-SLSC, GPU-SLSC is preferred over DAS when considering clinical photoacoustic-based visual servoing applications utilizing energies within existing laser safety limits.

The computation times shown in Fig. 4 suggest that real-time GPU-SLSC imaging is achievable for most clinical scenarios in which the photoacoustic source is located as deep as 10 cm, with a laser PRP of 100 ms or higher. Figure 4 shows that image depth d has the largest effect on the processing time (of the three parameters varied), as frame rate decreases by a factor of ∼2 when increasing d from 5 to 15 cm. While the feasibility of real-time imaging modalities depends on the amount of data to process, an imaging depth of 15 cm is uncommon for most interventional applications of photoacoustic imaging, including cardiac,^5^ abdominal,^70^ intravascular,^71^ hysterectomy,^72^ and spinal fusion surgery^51^^,^^73^ applications, suggesting that image depths as large as 15 cm are an unlikely concern for the real-time feasibility of GPU-SLSC in a majority of these cases. Nonetheless, the presented results at this 15-cm depth provide us with a worst-case scenario for system speed with internal light delivery and external ultrasound probe placement in these interventional applications.

The frame rate of 41.2 Hz (i.e., processing time of 24.3 ms) obtained with GPU-SLSC is either similar to or better than that obtained with GPU implementations of other advanced beamforming techniques. For example, real-time, 4-cm-deep DAS, delay-multiply-and-sum (DMAS), DAS + CF, or DMAS + CF imaging was achieved with processing times of 7.5, 7.6, 11.1, or 11.3 ms, respectively (with the exclusion of memory transfer times between the GPU and CPU).^43^ A variation of DMAS, namely the multiple DAS with enveloping beamformer, was implemented on a Quadro P5000 GPU to reconstruct 512×512 images in 41.62 ms.^74^ Another version of DMAS, proposed by Miri Rostami et al.,^75^ reported a 12-ms processing time for images of size 256×256  pixels. The reported GPU-SLSC frame rate is better than that achieved with these beamformers and with a parallel backprojection algorithm reporting 17-Hz frame rates when reconstructing 1024×512 duplex images.^76^ A more detailed comparison of processing speeds requires standardization of factors such as input data size, output image size, memory transfer evaluation, GPU hardware, and overhead.

Photoacoustic targets reconstructed with SLSC beamforming generally produced signals with different shapes when compared with targets reconstructed with DAS beamforming, as shown in Fig. 8(a). This change in signal morphology is caused by the degradation in axial resolution with the chosen axial kernel size of k=11 and the improved lateral resolution with the chosen M=25. Although an axial kernel size of k=11 was chosen to maximize the gCNR value between signal and background regions, the associated axial resolution degradation does not significantly affect the performance of our visual servoing algorithm, which tracks the displacement of the optical fiber in the lateral dimension. In addition, the target size reduction in the lateral dimension benefits the performance of the visual servoing algorithm, as the estimation accuracy of the target centroid is enhanced in this dimension. Therefore, the modified target size is either neutral to or beneficial for the clinical application of visual servoing.

One study limitation is that the number of scanlines and high-pass filter coefficients was not varied nor evaluated. However, we determined that a line density of 1 (i.e., 128 scanlines) was sufficient to visualize an optical fiber with a core diameter of 1 mm, considering that the resolution in the lateral dimension is approximately half the element pitch (i.e., 0.15 mm). Although an increased number of high-pass filter coefficients ensures the removal of DC components without compromising the frequency spectrum of the radio frequency signals of interest, in practice, the radio frequency signals are bandpass filtered to the operation range of the ultrasound probe before being stored in memory. Therefore, increasing the high-pass filter coefficient is not expected to affect the overall quality of the SLSC images.

Future work will advance implementation of GPU-SLSC beamforming for clinical visual servoing applications with the primary goal of reducing the risk of ionizing radiation exposure by substituting fluoroscopy usage with the proposed approach.^5^^,^^77^ For example, the *in vivo* setup described in our previous publication^5^ implemented visual servoing with DAS beamforming to guide a cardiac catheter tip using a fluence of $365.5\;\mathrm{mJ}/{\mathrm{cm}}^{2}$, which is higher than the $25.2\text{-}\mathrm{mJ}/{\mathrm{cm}}^{2}$ safety limit for skin at the same 750-nm wavelength.^65^ SLSC-based visual servoing has the potential to provide similar visual servoing performance to higher energy DAS results when using energies below existing safety limits, as indicated by the results in Fig. 10. In addition, the SNR enhancement provided with GPU-SLSC is expected to be beneficial in other applications with high noise and high acoustic scattering or attenuation, such as navigating inside bony anatomy during spinal fusion surgeries^51^ or endonasal transphenoidal surgeries^16^ and navigating within liver tissue during surgeries, biopsies, or radio frequency ablations.^70^^,^^78^

## 5 Conclusion

This paper presents the first known implementation of real-time SLSC beamforming for photoacoustic imaging, which was enabled by GPUs and parallel processing techniques. When selecting optimal beamforming parameters for visual servoing tasks, a factor of 348 speedup was achieved when compared with CPU-SLSC implementations. This speedup allows for real-time visualization of photoacoustic images for any laser with pulse repetition frequencies up to 41.2 Hz. *Ex vivo* results with bovine tissue and *in vivo* results from cardiac data demonstrate that GPU-SLSC imaging has the potential to enable visualization of photoacoustic signals obtained with low laser energies during photoacoustic-guided interventions, which is promising for the miniaturization of lasers to perform photoacoustic-based visual servoing in the operating room or interventional suite. In addition, GPU-SLSC imaging outperformed DAS imaging when jointly comparing probe centering, image segmentation, and fiber tracking tasks in the presence of low channel SNRs and when using laser energies that meet existing laser safety requirements.
